# Effects of a music therapy and music listening intervention for nursing home residents with dementia: a randomized controlled trial

**DOI:** 10.3389/fmed.2024.1304349

**Published:** 2024-02-06

**Authors:** Anna-Eva J. C. Prick, Sytse U. Zuidema, Peter van Domburg, Peter Verboon, Annemieke C. Vink, Jos M. G. A. Schols, Susan van Hooren

**Affiliations:** ^1^Department of Creative Arts Therapies, Zuyd Hogeschool, Heerlen, Netherlands; ^2^Department of Clinical Psychology, Open Universiteit, Heerlen, Netherlands; ^3^Department of Primary and Long-Term Care, University of Groningen, University Medical Center Groningen, Groningen, Netherlands; ^4^Alzheimer Centrum Groningen, Groningen, Netherlands; ^5^Department of Neurology, Zuyderland Medical Center, Heerlen, Netherlands; ^6^Department of Methods and Statistics, Open Universiteit, Heerlen, Netherlands; ^7^Department of Music Therapy, ArtEZ University of the Arts, Arnhem, Netherlands; ^8^Department of HSR, Maastricht University, Maastricht, Netherlands; ^9^Department of Family Medicine, Maastricht University, Maastricht, Netherlands

**Keywords:** dementia, music therapy, music listening, problem behaviour, quality of life, neuropsychiatric symptoms, psychosocial intervention, nursing home

## Abstract

**Introduction:**

The aim of the present study was to evaluate the effects of an individual music therapy intervention and an individual music listening intervention on neuropsychiatric symptoms and quality of life in people with dementia living in a nursing home and on professional caregiver’s burden to be able to make statements about their specific value of application in clinical practice.

**Methods:**

A multicenter single blind randomized controlled trial with three groups was performed: an individual music therapy intervention (IMTI) group (*n* = 49), an individual music listening intervention (IMLI) group (*n* = 56) and a control group (*n* = 53) receiving usual care. The interventions were given during three weeks, three times a week on non-consecutive days during 30–45 minutes for in total nine sessions. The endpoint of the study is the difference from baseline to interim (1,5 week), post-intervention (3 weeks) and follow-up (6 weeks) in reported scores of problem behaviour (NPI-NH) and quality of life (Qualidem) in people with dementia and occupational disruptiveness (NPI-NH) in care professionals.

**Results:**

In total 158 people with dementia were randomized to one of the two intervention groups or the control group. Multilevel analyses demonstrated that hyperactive behaviour assessed by the NPI-NH was significantly more reduced for the IMLI group at follow up and that restless behaviour assessed by the Qualidem was significantly more reduced for the IMTI group at post and follow-up measurement compared to the control group. No significant effects between groups were found in other NPI-NH clusters or Qualidem subscales.

**Conclusion:**

In conclusion, because we found no convincing evidence that the IMTI or IMLI is more effective than the other both interventions should be considered in clinical practice. For the future, we advise further research into the sustainability of the effects with alternative designs, like a single case experimental design.

## Introduction

Worldwide there are about 50 million people living with dementia and this number will triple in 2050 (1). Dementia is an umbrella term for a number of neurocognitive diseases characterized by progressive cognitive declines as well as behavioural alterations (2, 3). More than 80% of people with dementia develop neuropsychiatric symptoms (NPS) symptoms in the course of their disease, such as depression, apathy, agitation, delusions and anxiety (4–7). NPS appears to be the main factor affecting quality of life (QoL) of both people with dementia and their caregivers (6, 8). Furthermore, NPS are implicated in a cycle of negative events including placement in residential care, even risk of death in individuals with Alzheimer’s disease and high societal costs (9). The clinical presentation of NPS is determined by various disease specific, individual, psychosocial and contextual factors that require highly individualized psychosocial interventions supporting people’s existing capabilities (10–12). Such psychosocial interventions need to be safe and accessible and serve as a first step when problem behaviour occurs in people with dementia (13). Psychosocial interventions can greatly reduce the necessity of psychotropic drug therapies (14, 15).

Music (therapy) interventions are promising psychosocial interventions. The non-verbal nature of music provides a low-threshold approach, which can be offered up to people with dementia who have difficulty expressing themselves verbally. In the clinical setting, music is often used as an indirect intervention to improve the atmosphere and pleasure, often specified as reminiscence. But it can also be used as a personal directed psychosocial intervention specifically focusing on reducing NPS. During these interventions, abilities are addressed that are preserved in persons with dementia, such as music making, singing and moving on music (16). This is different from other therapeutic interventions, such as cognitive behaviour therapy and solution-based therapy, which rely on verbal and cognitive abilities to a higher degree (17). When music is therapeutically applied guided by a music therapist, it is called music therapy. A music therapist is specifically trained in psychotherapy through music and attunes continuously to the person and the behavioural and psychological symptoms (18). In its application, music therapy is divided into receptive music therapy and active music therapy (19). During receptive music therapy, a person with dementia is listening to music based on his/her personal preferences under the guidance of a music therapist. Meanwhile, during active music therapy a person with dementia is actively invited to play an instrument, singing, or creating a song. Active music therapy has been suggested to be more effective in decreasing problem behaviours than other types of music interventions (20). For both active and receptive music (therapy) interventions the use of individual preference of music is extremely important because pleasant and unpleasant music elicit different emotional responses (21) and the impact is influenced by the type of music used (22). Personalized music is defined as music that is integrated into one’s life and is based on personal preference (23). Listening to personalized music is widely available, easy to implement but considered no real therapy when it is not guided by a (music) therapist.

Several systematic reviews have shown that music (therapy) interventions with personal music preference are effective in reducing NPS such as depression, anxiety and agitation and increase quality of life (QoL). A Cochrane review (16) confirmed the positive effects of music therapy in dementia care on reducing depressive symptoms and improving overall behavioural problems. Recent systematic reviews (24, 25) suggest music therapy improves memory and verbal fluency, reduces anxiety and apathy, and has short-lasting effects on the quality of life of people with dementia. However, these reviews revealed no significant effects of music therapy on agitation and aggression. No long-term music therapy effects were reported as well (16, 24, 25).

There are many reviews available investigating the effects on music (therapy) on various dementia related symptoms and showing the therapeutic potential of music in dementia care. Nevertheless, intervention studies or high-quality trials that show effects on the broader range of NPS are scarce and charged with methodological restrictions (16, 24, 26–28). The design and implementation of this kind of research is faced with many practical and theoretical difficulties (26, 29, 30). Conclusions are limited by the small number of fully powered robust clinical trials. Small sample sizes were one of the main limitations of included studies in the reviews with low recruitment rates, often the cause of underpowered studies. And there are also studies that question the specific effects of music (therapy) on people with dementia (26). Furthermore, it is important to compare the effects of individual music therapy with an individual music listening intervention to be able to make statements about their specific value of application in clinical practice.

For this study, we developed in collaboration with experienced music therapists in dementia care an individual music therapeutic intervention (IMTI) guided by a professional music-therapist and an individual music listening intervention (IMLI) guided by a nurse. These developed interventions are specially aimed at reducing problem behaviour in nursing home residents with dementia. With the knowledge that randomized controlled trials (RCT) are still the gold standard of evaluating the effects of health interventions (31), we performed a multicenter single blind RCT to compare the developed interventions (IMTI and IMLI) with a control group receiving usual care. The aim of the present study was to evaluate the effects of IMTI and IMLI on neuropsychiatric symptoms and quality of life in people with dementia and on professional caregiver’s burden. Also, practical experiences of music therapists, nurses and informal caregivers with the developed IMTI and IMLI have been systematically evaluated in a process evaluation published elsewhere (32).

## Methods

### Design

This study concerned a multicenter single blind RCT with four measurements: baseline (T0), after one and a half weeks (interim measurement, T1), after 3 weeks (post intervention, T2), and after 6 weeks (follow-up, T3). This RCT included two music treatment interventions (IMTI and IMLI) and a control group. The control group received usual care including usual non-pharmacological interventions like physical exercise, reminiscence therapy or validation, but without any music component. Eligible participants were randomly allocated to receive the IMTI (*n* = 49), IMLI (*n* = 56) or usual care (*n* = 53). This RCT was reported following CONSORT guidelines for non-pharmacological treatment (33). Study visits occurred between July, 2017 and September, 2020. Alongside this RCT, we performed a process evaluation with qualitative research in which the experiences of music therapists, professional and informal caregivers with both interventions have been researched. The results of this process evaluation are published elsewhere (32).

### Procedure

For both intervention groups, a standardized treatment schedule was developed in which the supervised intervention was given next to usual care during 3 weeks, three times a week on non-consecutive days during 30–45 min in accordance with practicability in residential care. A logbook was kept by music therapists (for IMTI) and professional caregivers (for IMLI) in which intervention adherence was noted. The control group received usual care. The interventions were offered at varying times of the day in close coordination with the involved care team of the participant. The time of the assigned intervention was tailored to personal client objectives (e.g., activation or relaxation) and individual daily client schedules taking into account possible overload and agreements with family or other obligations.

All outcome measures were assessed by an independent trained research assistant who was blinded to the intervention. This independent research assistant visited the involved professional caregivers of the participants in the nursing home for the NPI-NH and Qualidem assessments. For each participating person with dementia, the questionnaires were systematically administered across all four measurements to the professional caregiver closely involved in the care for the respective participant.

### Participants

Participants were recruited from four nursing home organizations (spread over 12 locations) with expertise in dementia in the south of the Netherlands. Participants were eligible for inclusion when diagnosed with any type of dementia on admission by a professional (physician or psychologist) according to the Diagnostic and Statistical Manual of Mental Disorders, Fifth Edition, regardless the etiology (minimal Global Deterioration Scale score 2) (3, 34). Furthermore, participants were only included with one or more NPS observed by the attending physician or psychologist. Further inclusion criteria were a written informed consent by a responsible family member in accordance with local ethical committee regulations and a life expectancy for at least 2 months. Exclusion criteria were any somatic or not-dementia related cause of behavioural problems, delirium, deafness and change of any medication during the last 2 weeks before inclusion. In addition, treatment with psychotropic drugs was not a contradiction for inclusion, provided that it had not changed in the last 2 weeks before inclusion. For the recruitment, local clinical research coordinators and involved staff from the four participating nursing home organizations approached potential residents either face-to-face and/or by talking with the informal caregiver about the study. Eligible participants and their professional and informal caregivers were provided with detailed information of the study described in the information letter and explained orally by the principal investigator. Subjects could withdraw from the study at any time without any effect on their usual care.

### Sample size

We calculated the number of residents needed to detect a medium effect size with 90% power with a randomized complete-block design (with three groups and four measurements) at minimal 143 (35). Assuming a dropout rate of 20%, this leads to an inclusion of a total of 172 people with dementia.

### Randomization and blinding

A researcher not otherwise involved in the study performed randomization. Participants were informed that they would randomly be placed in one of the three groups, including a no treatment music group, with equal chance of being assigned to any group. To maintain independence between clinical and experimental data, clinicians (music therapists, psychologists and other professional caregivers) were not involved in data collection. Research personnel were blinded to treatment assignment. Randomization codes were computer generated in blocks of six participants.

### Interventions

#### Individual music therapy intervention (IMTI)

The IMTI was an active music therapy intervention (min. 30–max. 45 min three times a week for three weeks, for a total of nine sessions) using musical improvisation with music instruments or voice/singing and movement guided by a music therapist. Music therapists selected and applied musical elements (melody, rhythm, harmony and sound) adequately, tailored to a recipient’s individual needs and goals. Every session consisted of three phases: opening-main-closing. During the opening phase of every session, experience-oriented working was the starting point to get in touch with the resident and to create a sense of security (validation). In the main phase, the music therapist worked towards the reduction of physical and emotional tension (depending on the goal) using recognized techniques and improvisation. The intervention ended with a closing phase (goodbye) in which the music therapist took care of a relaxed transfer to daily care. After the closing phase, the resident was transferred by the music therapist to the nursing staff including musical advises what can be done for the resident to relax or to activate so that daily care can be built on the experiences of the music therapist. The music therapy session took place in a quiet (therapy) room (this may be the participant’s own room) where distracting stimuli were avoided. Six experienced and well-trained music therapists were available for the intervention at the different locations. Each participant received his or her music therapy session from the same music therapist. All involved music therapists had a bachelor’s degree in music therapy and had at least 1 year of experience in working with people with dementia. The IMTI was developed in collaboration with experienced music therapists, dementia care professionals and client representatives aimed at reducing or stabilizing of problem behaviour in dementia (32).

#### Individual music listening intervention (IMLI)

During the IMLI the personalized music was offered by an iPod, in a familiar quiet environment, supervised by a professional caregiver (during 30–45 min three times a week for 3 weeks, for a total of nine sessions). The IMLI was based on the guidelines developed for this purpose by Gerdner (23): (1) music selection according to patient’s personal preference. To find out personal music preferences, the involved professional caregiver used a standardized inventory instrument of personal music preferences, namely Assessment of Personal Music Preference Questionnaire (APMPQ) (36) and the person with dementia was interviewed by a professional caregiver (if possible), supplemented with an interview with close relatives (informal caregiver, child(ren) brothers/sisters/old friends); (2) music material file (iPod) preparation for each resident; (3) factors in the environment that may cause the person to be agitated should be avoided. The intervention should preferably be offered on residents’ room as a quiet and comfortable environment. The professional caregiver ensured that the person with dementia sits comfortably and explained that he/she was going to listen to music. Before putting headphones on his/her head, the professional caregiver tested the sound volume; (4) the professional caregiver monitored the intervention and regularly observed whether everything is going well and how the participant reacted to the music (in case of agitation, music listening was interrupted). When the person with dementia fell asleep and the music was not disturbing, it was considered an acceptable form of relaxation.

### Outcome measures

The primary outcomes were the Neuropsychiatric Inventory Nursing Home version (NPI-NH) scale to assess the dementia-related behavioural symptoms and the Qualidem as a patient-related outcome measure of the QoL (37, 38). A trained research assistant administered both measures to involved professional caregivers of the participants.

NPI-NH is a derived version of the NPI and validated for use in nursing homes and the Dutch version has been shown to be consistent with clinical taxonomy and relatively stable across dementia stages (39, 40). The NPI-NH consists of a semi-structured interview by which the severity and frequency of disturbances in 12 symptom domains is rated. Apart from the presence of a symptom (yes or no), the frequency (F) on a 4-point scale and the severity (S) on a 3-point scale of each behaviour are rated. The total score is calculated by summing the 12 F × S scores yielding a range of 0–144. Clinically relevant determinations or changes of a symptom are defined by a score of ≥4 points (41). A 5-point scale for professional caregivers’ occupational disruptiveness was developed separately, to allow an assessment of the impact of behavioural disturbances on professional caregivers. In accordance with the proposal of the European Alzheimer’s Disease Consortium (EADC) the neuropsychiatric symptoms were grouped into hyperactive (agitated), affective (caused by mood-changes), psychotic, and apathic behaviour clusters (42). The validity and interrater and test-retest reliability of the NPI-NH have been well established in several languages including Dutch (37, 41). The Cronbach’s alpha varied from 0.71–0.83 for NPI-NH frequency scores and from 0.73–0.81 for severity scores for T0, T1, T2 and T3 in this study.

The QUALIDEM has repeatedly been shown to be one of the most suitable QoL instruments to use for people with dementia in nursing homes (43). The original Dutch version of the QUALIDEM consists of 40 items describing observable behaviour in nine different subscales concerning relationships with staff (7) or other residents (6), positive or negative affect (9), restless behaviour (3), feeling at home (4), isolation (3), having something to do (2), positive self-image (3) and three additional questions (concerning eating and preferring to lie in bed). The four response options were never, seldom, sometimes, and often. The QUALIDEM has good reliability (38) and the Cronbach’s alpha varied from 0.84 to 0.89 for T0, T1, T2 and T3 in this study.

Secondary outcomes concerned the Cantril’s ladder (44) to assess well-being, the observational instrument “Kommunikationsfahigkeit bei demenzkranken Menschen” (KODEM) to assess communication behaviour and the observational instrument positive response scale (PRS) to assess well-being (45, 46). The results of the analyses of the Cantril’s ladder, in which the person with dementia used a visual scale to indicate how he/she feels immediately before and after each session, are published in our process evaluation (32). The correct interpretation of the observations of the observational scales CODEM and PRS proved to be very complicated in practice. In the PRS, for example, the emotion crying is scored negatively, while during music therapy, crying can be a positive emotion for someone with dementia who has difficulty showing emotions. The goal of music therapy may be to give space to someone’s grief or emotions. Because the complexity of analyzing the observational data, the data requires thorough study in order to properly interpret its validity and psychometric properties. The results will be published elsewhere.

### Statistical analysis

First, descriptive statistics were computed for the key variables (NPI-NH and Qualidem) and important background variables (age, gender, Global Deterioration Scale score, dementia type, educational level) (Table 1). Differences between the experimental groups of the key variables at baseline for the background variables were checked using the *F*-test (ANOVA). The data were then reshaped to be in long-format, making each record correspond to a measurement. In the long-format data of the measurements were nested within the subjects.

**Table 1:** Baseline characteristics of people with dementia.

	IMTI (*n* = 49)	IMLI (*n* = 56)	CONTROL (*n* = 53)	THE WHOLE SAMPLE (*N* = 158)	*p*-value (2-sided)
Age, years, mean (SD)	81.7 (7.6)	81.8 (9.3)	82.3 (9.9)	81.9 (9.0)	0.93
Gender, *n* (%)					0.05
Females	30 (61.2)	43 (76.8)	29 (54.7)	102 (64.6)	
Males	19 (38.8)	13 (23.2)	24 (45.3)	56 (35.4)	
Global Deterioration Scale (GDS), *n* (%)					0.64
No cognitive decline	0 (0.0)	0 (0.0)	0 (0.0)	0 (0.0)	
Very mild cognitive decline	1 (2.1)	0 (0.0)	1 (1.9)	2 (1.3)	
Mild cognitive decline	2 (4.2)	2 (3.6)	0 (0.0)	4 (2.5)	
Moderate cognitive decline	3 (6.3)	4 (7.1)	2 (3.8)	9 (5.7)	
Moderately severe cognitive decline	14 (29.2)	20 (35.7)	12 (22.6)	46 (29.3)	
Severe cognitive decline	14 (29.2)	22 (39.3)	22 (41.5)	58 (36.9)	
Very severe cognitive decline	7 (14.6)	0 (0.0)	0 (0.0)	7 (4.5)	
Mixed/changing GDS	7 (14.6)	8 (14.3)	16 (30.2)	31 (19.7)	
Dementia type, *n* (%)					0.55
Alzheimer dementia	23 (54.8)	25 (51.0)	21 (51.2)	69 (52.3)	
Vascular dementia	6 (14.3)	10 (20.4)	3 (7.3)	19 (14.4)	
Lewy body dementia	1 (2.0)	0 (0.0)	0 (0.0)	1 (0.8)	
Frontotemporal dementia	3 (7.1)	2 (4.1)	3 (7.3)	8 (6.1)	
Korsakov dementia	2 (4.8)	1 (2.0)	2 (4.9)	5 (3.8)	
Young onset dementia	0 (0.0)	0 (0.0)	1 (2.4)	1 (0.8)	
Parkinson dementia	2 (4.8)	1 (2.0)	0 (0.0)	3 (2.3)	
Education, *n* (%)					0.19
<6 years low education	0 (0.0)	0 (0.0)	1 (5.0)	1 (1.5)	
6 years low education	1 (5.6)	2 (7.4)	3 (15.0)	6 (9.2)	
High school	1 (5.6)	7 (27.9)	3 (15)	11 (16.9)	
Intermediate vocational education	10	14	9	33 (20.9)	
Bachelor degree	5 (27.8)	4 (14.8)	2 (10.0)	11 (16.9)	
Master degree	1 (5.6)	0 (0.0)	2 (10.0)	3 (4.6)	
NPI-NH, mean (SD)					0.05
NPI-NH cluster psychotic (domain delusions + hallucinations)	2.6 (5.1)	4.0 (6.3)	2.6 (5.6)	3.1 (5.7)	0.38
NPI-NH cluster affective (domain depression + anxiety)	3.4 (4.2)	3.3 (4.9)	4.6 (5.7)	3.8 (5.0)	0.33
NPI-NH cluster apathic (domain apathy)	3.0 (4.0)	2.4 (3.5)	2.8 (4.0)	2.8 (3.8)	0.70
NPI-NH cluster hyperactive (domain irritability + disinhibition + agitation + aberrant motor behaviour)	11.7 (9.8)	18.0 (11.1)	11.3 (12.7)	13.7 (11.6)	0.00
NPI-NH total score	25.4 (19.9)	34.6 (18.6)	26.3 (22.7)	28.9 (20.8)	0.05
Occupational disruptiveness score	10.5 (8.2)	12.9 (7.0)	10.3 (8.2)	11.3 (7.8)	0.19
QUALIDEM, mean (SD)					
A: Care relationship	14.2 (5.2)	13.5 (4.8)	15 (6.33)	14.2 (5.5)	0.37
B: Positive affect	13.9 (3.5)	14.9 (3.2)	14.1 (4.0)	14.3 (3.6)	0.34
C: Negative affect	6.1 (2.2)	5.9 (2.7)	5.7 (2.5)	5.9 (2.5)	0.70
D: Restless behaviour	5.2 (2.4)	4 (3.0)	5 (3.1)	4.7 (2.9)	0.08
E: Positive self-image	7.4 (2.5)	7.7 (2.3)	7.1 (2.2)	7.4 (2.4)	0.47
F: Social relations	11.1 (3.8)	10.8 (3.8)	10.3 (4.0)	10.7 (3.9)	0.60
G: Social isolation	6.8 (2.3)	5.7 (2.4)	6.5 (2.4)	6.3 (2.3)	0.06
H: Feeling at home	10 (2.5)	10.1 (2.1)	9.5 (2.5)	9.9 (2.4)	0.50
I: Something to do	2.2 (2.1)	2.4 (2.2)	2.1 (1.7)	2.3 (2.0)	0.76

SD, standard deviation; IMTI, individual music therapy intervention; IMLI, individual music listening intervention; NPI-NH, Neuropsychiatric Inventory-Nursing Home version.

Next, multilevel analyses (MLA) (47) were performed with the package Ime4 (48) in R (49), to explore the effects of the interventions on the various subscales of the Qualidem and the NPI-NH total score and cluster scores (clusters: hyperactive, psychotic, affective, apathic and occupational disruptiveness). MLA deals with nested data, and in this study time points are nested within subjects. Multilevel analysis is an alternative for repeated measures (RM) ANOVA, which has several advantages. The assumptions underlying MLA are less strict than for RM-ANOVA. Contrary to RM-ANOVA MLA can analyse subjects with missing data at one or more time points which makes MLA more efficient. MLA distinguishes between fixed and random effects. In our model we specified one random effect: the intercept, which means we allow the intercepts to vary across subjects. This implies that we expect that subjects differ in the value of the dependent variable at T0. The intra class correlation (ICC) indicates how much variance can be attributed to the subjects (50). In the results we focus on the fixed effects which can roughly be interpreted as regression coefficients. In addition, a per protocol analysis was performed in which only people were selected who attended five sessions or more in the IMTI and IMLI.

### Ethical considerations

The study protocol was approved by the Dutch Medical Ethical Committee (METC No. 17-T-30) and registered at the International Clinical Trials Registry Platform (ICTRP) (Identifier: NL8861).

## Results

### Baseline data

**Figure 1:**
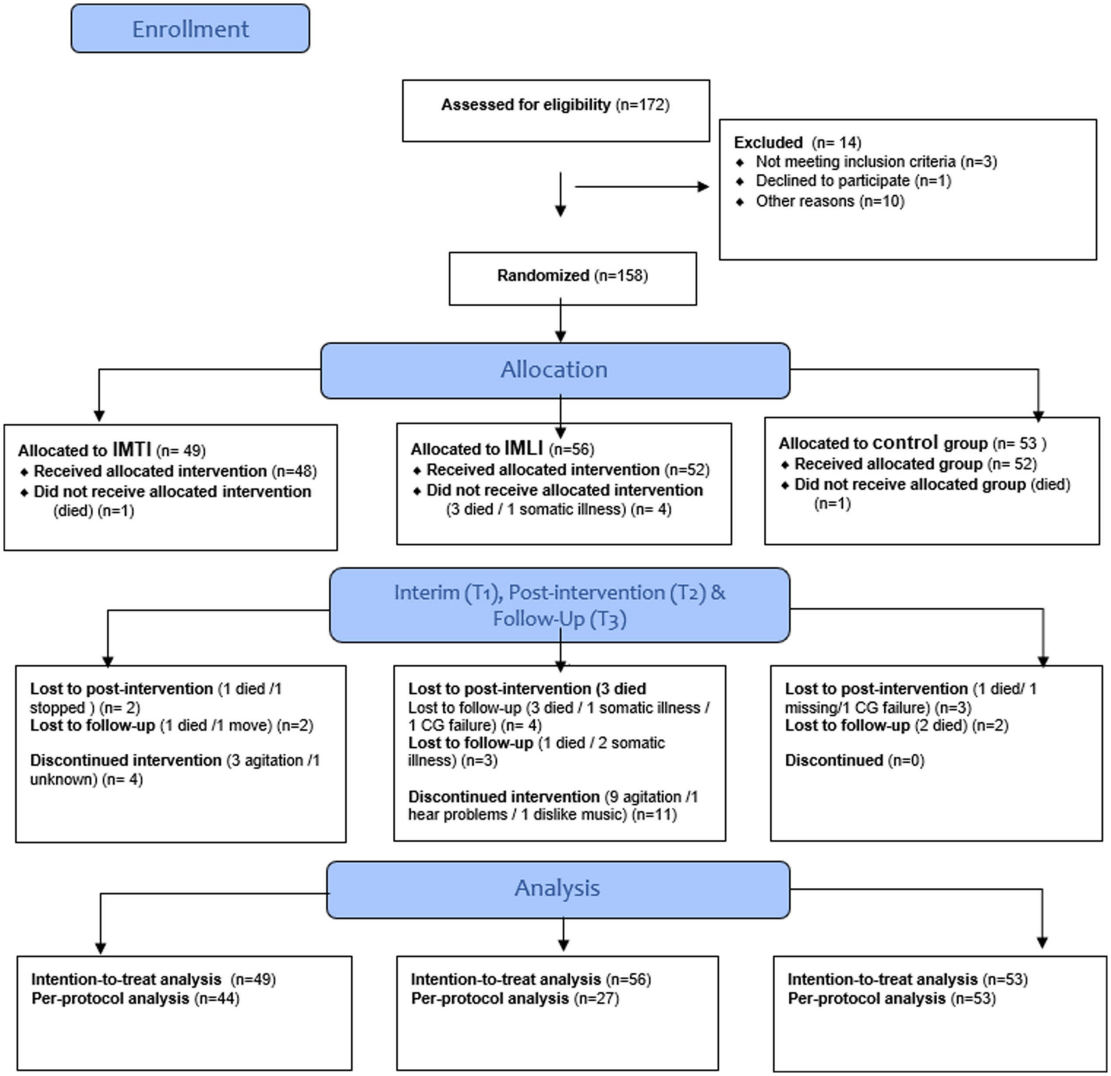
CONSORT flow diagram.

The CONSORT diagram in Figure 1 details the subject selection and allocation procedures. In total 172 people with dementia were assessed for eligibility of which 14 people with dementia were excluded. In total 158 people with dementia were randomly assigned to the IMTI group (*n* = 49), IMLI group (*n* = 56) or control group (*n* = 53). The dropout rate from baseline to 3 weeks post-intervention was 5.7% (9 of 158) and the dropout rate from 3 to 6 weeks follow-up was 4.4% (7 of 158). Attrition analyses of subjects that completed the study compared to participants that discontinued the study showed no significant differences in background variables (*p* > 0.05). The treatment compliance differed between the two intervention groups: in total 44 participants allocated to the IMTI group, and 27 participants allocated to the IMLI group followed 5–9 sessions (Table 2).

**Table 2:** Frequencies per condition.

	Full sample (intention to treat analysis)	Followed all 9 sessions	Followed ≥5 sessions (per protocol analysis)
Control	53	53	53
IMTI	49	34	44
IMLI	56	15	27
Total	158	102	124

### Multilevel analyses on NPI-NH

**Figure 2:**
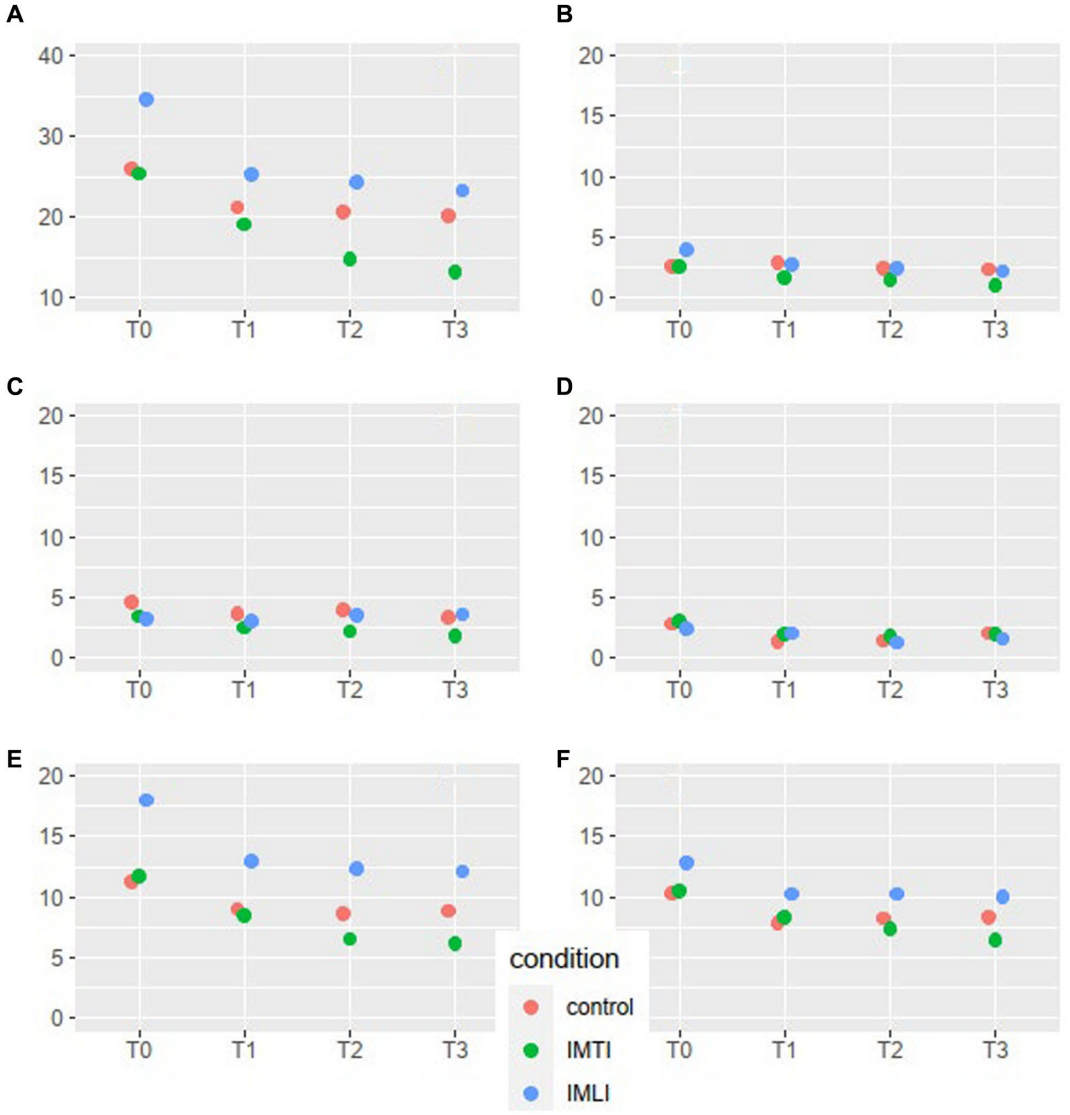
NPI-NH total score and cluster means per condition and time point for all subjects. The scales of the NPI-NH are: A = total score, B = cluster psychotic, C = cluster affective, D = cluster apathic, E = cluster hyperactive, F = occupational disruptiveness.

The results of the MLA’s on the NPI-NH total and cluster scores are given in Figure 2. All groups, including the control group, showed a statistically significant reduction of the total NPI-NH score at T1, T2 and T3 (Table 3). As shown in Figure 2, the results of the MLA’s on the NPI-NH clusters, especially for the outcome clusters hyperactive and occupational disruptiveness, visually indicate a reduction of both intervention groups at T2 and T3 in comparison with the control group. For the cluster hyperactive, this reduction is statistically significant for the IMLI group at T3 (*p* = 0.03) (Table 4). The ICC is 0.64 for the NPI-NH.

**Table 3:** Multilevel analyses NPI-NH total score.

	Estimate	SE	df	*t*	*p*	95% CI lower limit	95% CI upper limit
(Intercept)	26.294	2.77	250.3	9.50	0.000	20.91	31.68
IMTI	−0.898	3.97	250.3	−0.23	0.821	−8.63	6.84
IMLI	8.321	3.89	250.3	2.14	0.034	0.74	15.90
TimeT1	−5.034	2.31	422.6	−2.18	0.030	−9.52	−0.55
Timet2	−5.747	2.31	422.9	−2.49	0.013	−10.23	−1.26
TimeT3	−5.923	2.31	422.9	−2.57	0.011	−10.41	−1.44
IMTI:timeT1	−1.368	3.31	421.7	−0.41	0.680	−7.80	5.07
IMLI:timeT1	−4.637	3.26	422.7	−1.42	0.155	−10.96	1.69
IMTI:timeT2	−4.052	3.29	421.7	−1.23	0.219	−10.45	2.34
IMLI:timeT2	−4.674	3.23	422.4	−1.45	0.148	−10.94	1.59
IMTI:timeT3	−5.646	3.31	422.1	−1.70	0.089	−12.09	0.79
IMLI:timeT3	−5.144	3.26	422.9	−1.58	0.115	−11.47	1.19

**Table 4:** Multilevel analyse NPI-NH cluster E: hyperactive.

	Estimate	SE	df	*t*	*p*	95% CI lower limit	95% CI upper limit
(Intercept)	11.275	1.50	228.5	7.53	0.000	8.36	14.19
IMTI	0.434	2.15	228.5	0.20	0.840	−3.76	4.62
IMLI	6.725	2.11	228.5	3.19	0.002	2.62	10.83
TimeT1	−2.161	1.14	421.8	−1.89	0.059	−4.38	0.06
TimeT2	−2.636	1.14	422.2	−2.31	0.022	−4.86	−0.41
TimeT3	−2.327	1.14	422.2	−2.04	0.042	−4.55	−0.11
IMTI:timeT1	−1.035	1.64	421.1	−0.63	0.528	−4.22	2.15
IMLI:timeT1	−2.987	1.61	422.0	−1.85	0.065	−6.12	0.15
IMTI:timeT2	−2.614	1.63	421.1	−1.60	0.109	−5.78	0.55
IMLI:timeT2	−2.946	1.60	421.7	−1.84	0.066	−6.05	0.16
IMTI:timeT3	−3.050	1.64	421.4	−1.86	0.064	−6.24	0.14
IMLI:timeT3	−3.450	1.61	422.1	−2.14	0.033	−6.59	−0.31

### Multilevel analyses on Qualidem

**Figure 3:**
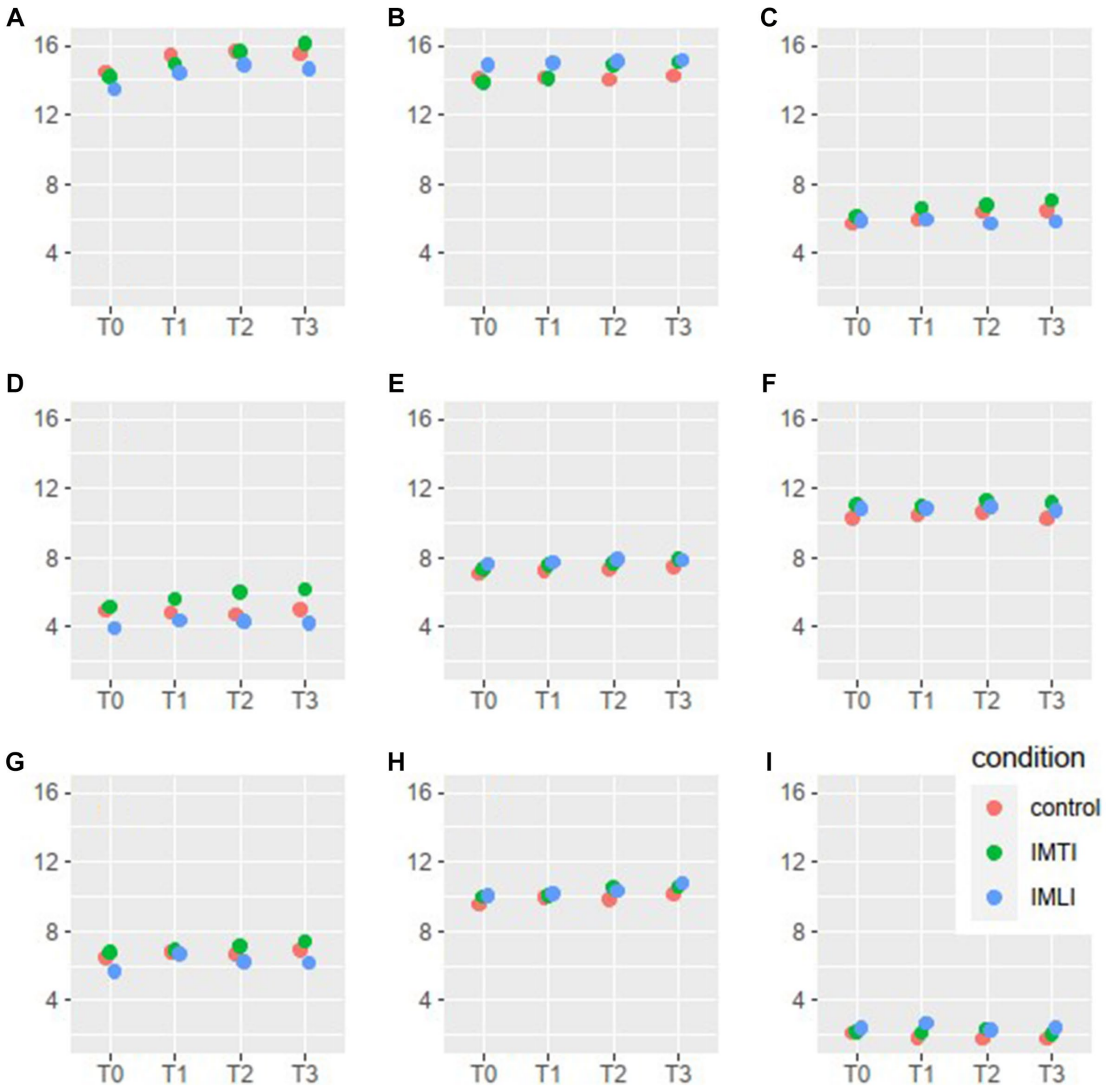
Qualidem subscales means per condition and time point. The 9 subscales of the Qualidem are A = care relationship, B = positive affect, C = negative affect, D = restless behaviour, E = positive self-image, F = social relations, G = social isolation, H = feeling at home, I = something to do.

**Figure 4:**
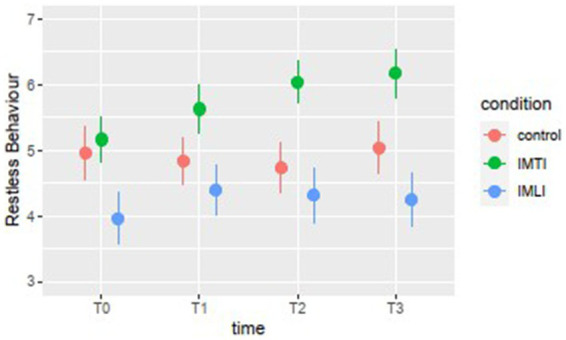
Subscale means “Restless behaviour” Qualidem.

The results of the MLA’s on the Qualidem subscales are given in Figure 3. The IMTI group showed, in comparison with the IMLI and control group, visually improved scores of the subscale outcomes care relationship, positive affect, negative affect, restless behaviour and social isolation at T2 and T3. For the subscale restless behaviour, this concerns a statistically significant improvement at T2 (*p* = 0.00) and T3 (*p* = 0.01) for the IMTI group (Table 5). This effect is illustrated in Figure 4. The vertical lines represent the confidence intervals around the predictions. The ICC is 0.77 for the Qualidem.

**Table 5:** Multilevel analyse Qualidem subscale D: restless behaviour.

	Estimate	SE	df	*t*	*p*	95% CI lower limit	95% CI upper limit
(Intercept)	4.961	0.40	210.7	12.52	0.000	4.19	5.73
IMTI	0.206	0.57	210.7	0.36	0.718	−0.90	1.31
IMLI	−0.999	0.56	210.7	−1.79	0.075	−2.09	0.09
Timet1	−0.171	0.27	423.1	−0.63	0.529	−0.70	0.36
TimeT2	−0.281	0.27	423.6	−1.02	0.306	−0.81	0.25
TimeT3	0.028	0.27	423.6	0.10	0.920	−0.51	0.56
IMTI:timeT1	0.681	0.39	422.4	1.74	0.082	−0.08	1.44
IMLI:timeT1	0.646	0.39	423.3	1.68	0.094	−0.10	1.39
IMTI:timeT2	1.223	0.39	422.7	3.13	0.002	0.46	1.98
IMLI:timeT2	0.664	0.38	423.2	1.73	0.084	−0.08	1.41
IMTI:timeT3	1.040	0.39	422.9	2.64	0.008	0.28	1.80
IMLI:timeT3	0.305	0.39	423.5	0.79	0.431	−0.45	1.06

Higher positive scores refer to less restless behaviour.

### Multilevel per protocol analyses

Per protocol analyses,^1^ in which only people were selected who attended five sessions or more in the IMTI (*n* = 44) and IMLI group (*n* = 27) showed no major differences or different significant effects compared to the intention-to-treat analysis.

## Discussion

The results of this multicenter single blind RCT (*N* = 158) showed some beneficial effects of the intervention groups (IMTI and IMLI) on neuropsychiatric symptoms and quality of life in people with dementia. For the total score of NPI-NH, the IMLI group showed a statistically significant reduction for the cluster “hyperactive” at follow-up measurement (T3). Furthermore, the IMTI group showed a significant improvement of the Qualidem subscale “restless behaviour” at post measurement (T2) and this effect persisted for 3 weeks after the intervention (T3).

Our results are partly in line with results of previous studies, in which music therapy and other music interventions for dementia have been shown to improve QoL and NPS, but effects on which specific NPS and QoL outcomes differ. The latest published Cochrane review (16) reported moderate-quality evidence that the music-based therapeutic interventions reduce depressive symptoms and no or low-quality evidence that the interventions may improve quality of life or decrease agitation. Previous studies on the specific treatment of depression have shown that a longer treatment duration than 3 weeks is needed, what might explain that we found no specific significant effects on the outcome NPI cluster “affective” in our study. A systematic review from Lam et al. (24) reports significant effects of both active and passive music therapy (music listening) on the outcomes anxiety, depression, quality of life and apathy. When music listening was the primary intervention they also reported significantly reduced agitation.

When focusing specifically at studies comparing active versus receptive music based interventions also varying results are found on NPS. In people with mild-to moderate dementia, Sarkämo et al. (51, 52) found that although both types of music intervention (singing and music listening) are effective for depressive symptoms, the pattern of improvement may be different between them. In moderate-severe dementia, Sakamoto et al. (53) reported that both kinds of intervention (active and receptive) have relaxing effects by parasympathetic activation, but active music intervention caused a greater reduction in behavioural problems. Raglio et al. (54, 55) reported higher effects of active music therapy than music listening on behavioural symptoms although the results did not reach statistical significance. More recently, Gómez-Gallego et al. (56) showed, when comparing active group music intervention versus group music listening in people with dementia, that active music therapy may improve behaviour (measured with NPI) of mild-to-moderate dementia residents. Instead, music listening had only a stabilizing effect on behaviour compared to the control intervention. However, a meta-analysis concluded that receptive music interventions may be more effective than active music therapy interventions in reducing anxiety, agitation, and other behavioural symptoms (57).

It is difficult to compare our results to other studies, because of limitations of the studies included in the cited reviews and meta-analyses because of low participant numbers, lack of standardized music therapy and high heterogeneity in outcome measures used. Furthermore, the comparison of our study results with other studies focusing on music interventions is difficult due to heterogeneous study designs, variations in duration and frequency of the studied music interventions, variations in supervisor (for example well educated music therapist or a (in)formal caregiver), variations in setting (group of individual sessions), variations in living environment of the participants (living in a nursing home or living at home) and variations in dementia stages and types. At the same time, this heterogeneity in population, outcome and intervention serves a purpose in clinical practice, in which a psychosocial intervention such as a music (therapy) intervention must be tailored to the needs of people with dementia and their caregivers.

### Strengths and limitations

A main strength is that that the planned sample size was achieved with adequate power. Small sample sizes or low recruitment rates often limit the success of comparable research studies (58), approximately 50% of clinical trials fall short of reaching their recruitment targets (59). Results of a recent review from Baker et al. (58), with the aim of researching the percentage of music therapy studies of people living with dementia met their target sample size, showed that only one study from the 14 studies reviewed (music therapy delivered in people with dementia living in nursing homes) reached 89% of its target sample size. Only five studies had a RCT design and only one study had a sample size over 100 (*N* = 117). Other sample sizes concerned less than 50 participants in total. At the same time, this power has to be seen in relation to treatment compliance, which was low for the IMLI group in our study. However, per protocol analyses, in which only people were selected who attended five sessions or more in the IMTI and IMLI group showed no different results compared to the intention-to-treat analysis. With the knowledge that recruitment in nursing homes is complex, challenging, and needs thorough planning (60), during the preparation of this RCT a lot of effort has been put by the research team into personal contacts with care organizations (from work floor to management). In each residential care organization, a local clinical research coordinator was appointed as a linking pin between clients, practitioners and policy makers, mostly a psychologist.

Another strength of our study concerned that the IMTI and IMLI were consensus-based developed together with music therapists specialized in dementia care and that the IMTI and IMLI were highly detailed described (32). In a recent study, Hakvoort and Tönjes (61) concluded that a lack of a clear description of the used intervention determines the inclusive effects of music (therapy) interventions described in systematic reviews and meta-analyses. Hakvoort and Tönjes presented three possible solutions to bridge the gap between research outcomes and practice: Create highly specific and detailed described music (therapy) interventions, develop consensus-based music therapy interventions (62) and to better define, understand, and formulate music (therapy) interventions using a shorter time frame. Regarding this last solution, most music (therapy) interventions have a duration of three to 6 months (63, 64). However, the results of these studies show that it is difficult to ascertain that change resulted solely from that music (therapy) intervention, as there were too many confounding factors (61). The IMTI and IMLI had a compact duration of 3 weeks with a high frequency of sessions (three times a week). This compact duration could also be raised as a limitation, but during the development of the interventions for this study this duration and high frequency were recommended by experienced music therapists working in elderly care. As far as we know, investigating the effects of high-frequency music therapy has not been studied before.

Nevertheless, there were several limitations. A limitation concerned that the IMLI intervention in practice was offered less often than prescribed according to protocol (9 sessions); also, the aim of the IMLI intervention protocol was to carry out the intervention by the same professional involved carer (IMLI) for each participant, but in practice this often proved to be unfeasible. To offer the IMLI according protocol was not always feasible in clinical practice because of changing duty schedules, staff movement, illness and work pressure among professional caregivers. These limitations did not apply to the IMTI, because the IMTI was offered by external independent music therapists who were not burdened with internal workload or changing duty schedules. Furthermore, the involved professional caregivers did not always keep track of which interventions and/or activities the control group received. As a result, there was not a complete overview of the care received in the control group. However, the control group did not receive any music(therapy) intervention or activity at all. In addition, it was not possible to double blind the study because the allocation of participants to the IMTI and IMLI could not be blinded from either the participant or the involved music therapists and professional caregivers. On the other hand, research assistants were blinded to treatment assignment. Completely controlling the environment and all confounding factors is often not possible in a nursing home environment and therefore it is important to be aware of these limitations and not distracted by them, because a single blind design is often the only option in this kind of research (60, 65).

### Future directions for clinical practice

Alongside this RCT, we performed a process evaluation with qualitative research in which the experiences of music therapists, professional and informal caregivers with both interventions have been researched. The results of this qualitative evaluation (32) showed that it is important that music therapists are involved in composing personalized music playlists and that music therapists can coach/supervise professional and informal caregivers in offering a music listening intervention. Both carers and music therapists recommended that experiences and gained insights from music therapists during the music therapy sessions have to be integrated into the IMLI by the involved music therapists and transferred under supervision to the (in)formal caregiver. For example, which music preference is for a person with dementia in which situation the best to reduce problem behaviour and at what times a listening intervention can best be offered to a specific person/situation. The results of this RCT showed some beneficial effects of both intervention groups (IMTI and IMLI), where the IMTI appears to be slightly more effective in reducing restless behaviour (Qualidem) and the IMLI appears to be slightly more effective in decreasing hyperactive behaviour (NPI-NH). By combining both the IMTI and IMLI interventions (IMLI as an extension of the IMTI intervention), the music therapy intervention can be continued outside the music therapy sessions (for example, during difficult situations in which problem behaviour arises) by informal caregivers under supervision of a music therapist at a distance. Perhaps a cumulative effect of both interventions can then be achieved in clinical practice. Although this was not investigated in this trial.

### Future directions for research

Various types of rating scales are used to measure the effects of music (therapy) interventions in people with dementia on NPS (for example NPI (different versions), CMAI, BEHAVE-AD and Global Depression Scale), and quality of life (for example QOL-AD, DQOL, Barthel Index, CBSQoL) (66). This makes it impossible to compare results between studies, and it may prevent from establishing evidence. For future research, to contribute to the accumulation of evidence for music therapy and to conduct good meta-analyses, it is important to standardize the rating scales. A standardized core outcome set (COS), consensus-derived, standardized, and parsimonious collection of outcomes to be reported at minimum in music (therapy) studies for people with dementia, can help to establish evidence in clinical research (67–70). However, there is no COS for music therapy for dementia currently; it will help if future research focuses on composing a COS.

Furthermore, for future research it’s necessary to get more insight into the mechanisms of evidence-based music (therapy) interventions on reducing NPS in persons with dementia living in nursing homes. This will allow a more personalized approach in reducing NPS and a better prediction and monitoring the effects of these interventions for future large-scale clinical studies. At this moment, specific mechanisms that may explain these effects in persons with dementia are mostly based on theoretical insights such as down or up regulating tempo of the music or tempo has effect on arousal regulation by and moving to rhythm of music which will ameliorate positive affect (71). Empirical studies examining the mechanisms of music (therapy) interventions in persons with dementia are scarce.

In addition, we would like to stress the importance of a mixed method design (quantitative and qualitative data collection) when conducting an effectiveness study of a psychosocial intervention like a music (therapy) intervention. Information gathered through qualitative methods, in addition to the quantitative data of a RCT, contributes to valuable insights for the implementation of an intervention. Qualitative research can assist in understanding the meaning and active mechanisms of an intervention to clients as well as clients’ beliefs about the treatment and expectations of the outcome. Besides, qualitative research is helpful in developing appropriate outcome measures for music therapy interventions. This is in line with a review, aimed at exploring what existing qualitative studies reveal about the implementation, effects and processes of psychosocial interventions for dementia (72).

Furthermore, we see a trend in which researchers are searching for alternatives to “randomized controlled trials” (RCTs), the gold standard in research (73). The search for such alternatives is especially interesting in complex interventions, which often consist of multiple components, often focus on multiple behaviours, require expertise and skills from those offering them and that take place in a rapidly changing reality over which the researcher does not always have influence (74). These characteristics of complex interventions are very recognizable for arts therapies interventions and for interventions for vulnerable people, like people with dementia. That is why alternatives are sought and applied within clinical research, like the ‘single case experimental design’ (SCED). The SCED is a pragmatic design that allows effects to be measured with a small number of participants (approximately 10–15). Repeated measurements per participant before, during and after the intervention provide insight into the effectiveness of an intervention. The participant then serves as control for himself. A gradual design, in which the intervention starts at a different time for the different participants, takes more into account that the results can be attributed to the intervention rather than influences from the context. A mixed method approach and the search for alternative designs shows that complex interventions for vulnerable people in the future can be investigated not only according to the classical RCT method but also with alternative, perhaps more suitable, designs.

Finally, there is an increasing tendency wherein people with dementia are remaining at home as long as possible. In the last decade, the proportion of people with dementia living in nursing homes has begun to decline in Western European countries, consistent with policy initiatives to provide care at home where possible in the face of growing numbers of people living with dementia (75). So, there is a great need for psychosocial interventions reducing problem behaviour and improving quality of life of people living with dementia at home. The IMTI and IMLI might also have potential to reduce problem behaviour and improve QoL for home living people with dementia and their caregivers. For future research it’s also worthwhile to study the effects of IMTI and IMLI for home living people with dementia.

## Conclusion

Music (therapy) interventions should be considered in dementia care in case of problem behaviour. Since there is no convincing evidence to suggest that one form of music-based intervention is more effective than the other, both individual active music therapy and individual receptive music listening interventions could be considered in clinical practice. This is in line with the NICE guidelines (76) and the Dutch guideline “Problem behaviour in people with dementia” which advise to start with a non-pharmacological treatment Zuidema and Smallbrugge (13), such as music therapy (77).

For the future, we advise further research into the sustainability of the effects and the differences between IMTI and IMLI, also in connection with the question of whether you should do IMLI as standard and IMTI for a selected group and/or combine both interventions to see if there is a cumulative effect. In addition, for a complex intervention in vulnerable people we advise to experiment with alternative, perhaps more suitable designs like the SCED for music (therapy) interventions in people with dementia, so that fewer large samples are needed.

## Data availability statement

The raw data supporting the conclusions of this article will be made available by the authors, without undue reservation.

## Ethics statement

The studies involving humans were approved by Dutch Medical Ethical Committee (METC No. 17-T-30). The studies were conducted in accordance with the local legislation and institutional requirements. Written informed consent for participation in this study was provided by the participants' legal guardians/next of kin.

## Author contributions

A-EP: Writing – original draft, Writing – review & editing. SZ: Writing – original draft, Writing – review & editing. PD: Writing – original draft, Writing – review & editing. PV: Formal analysis, Methodology, Writing – review & editing. AV: Writing – original draft, Writing – review & editing. JS: Writing – original draft, Writing – review & editing. SH: Writing – original draft, Writing – review & editing.

## Footnotes

^1^Per protocol analyses on request available via first author.
