# Supplementary material for: Effects of a music therapy and music listening intervention for nursing home residents with dementia: a randomized controlled trial
Source: Front Med (Lausanne). 2024 Feb 6;11:1304349. doi: 10.3389/fmed.2024.1304349 (PMC10877716; doi:10.3389/fmed.2024.1304349)
Supplement: Supplementary file 1 [file Data_Sheet_1.pdf]

Supplementary material

Correlation table for NPI-NH

	F	S	OD	FxS Total	FxS Psychotic	FxS Affective	FxS Apathic	FxS Hyperactive
F	1.00	0.93	0.85	0.95	0.60	0.56	0.40	0.82
S	0.93	1.00	0.92	0.94	0.61	0.59	0.39	0.79
OD	0.85	0.92	1.00	0.88	0.56	0.51	0.33	0.75
FxS Total	0.95	0.94	0.88	1.00	0.63	0.60	0.43	0.86
FxS Psychotic	0.60	0.61	0.56	0.63	1.00	0.35	0.18	0.36
FxS Affective	0.56	0.59	0.51	0.60	0.35	1.00	0.29	0.31
FxS Apathic	0.40	0.39	0.33	0.43	0.18	0.29	1.00	0.25
FxS Hyperactive	0.82	0.79	0.75	0.86	0.36	0.31	0.25	1.00

Notes: F=Frequency / S=Severity / OD=Occupational Disruptiveness

Correlation table for Qualidem

	A	B	C	D	E	F	G	H	I
A	1.00	0.32	0.08	0.24	0.15	0.19	0.48	0.28	0.06
B	0.32	1.00	0.20	0.22	0.23	0.61	0.25	0.17	0.42
C	0.08	0.19	1.00	0.43	0.26	0.16	0.28	0.11	0.16
D	0.24	0.22	0.43	1.00	0.11	0.24	0.49	0.09	0.20
E	0.15	0.23	0.26	0.11	1.00	0.03	0.20	0.27	0.02
F	0.19	0.61	0.15	0.24	0.03	1.00	0.32	-0.01	0.57
G	0.48	0.25	0.28	0.49	0.20	0.32	1.00	0.12	0.20
H	0.28	0.17	0.11	0.09	0.27	-0.01	0.11	1.00	-0.07
I	0.06	0.42	0.16	0.20	0.02	0.57	0.20	-0.07	1.00
